# A Biophysical Analysis on the Arm Stroke Efficiency in Front Crawl Swimming: Comparing Methods and Determining the Main Performance Predictors

**DOI:** 10.3390/ijerph16234715

**Published:** 2019-11-26

**Authors:** Ricardo Peterson Silveira, Susana Maria Soares, Rodrigo Zacca, Francisco B. Alves, Ricardo J. Fernandes, Flávio Antônio de Souza Castro, João Paulo Vilas-Boas

**Affiliations:** 1 Aquatic Sports Research Group, Universidade Federal do Rio Grande do Sul, Porto Alegre 90040-060, Brazil; ricardo@rpsportscience.com (R.P.S.); souza.castro@ufrgs.br (F.A.d.S.C.); 2 Faculty of Sport, CIFI2D and Porto Biomechanics Laboratory (LABIOMEP-UP), University of Porto, 4099-002 Porto, Portugal; susana@fade.up.pt (S.M.S.); rzacca@fade.up.pt (R.Z.); ricfer@fade.up.pt (R.J.F.); 3 Faculty of Human Movement Studies, University of Lisbon, 1649-004 Lisboa, Portugal; falves@fmh.ulisboa.pt

**Keywords:** Froude efficiency, propelling efficiency, economy, performance prediction

## Abstract

*Purpose:* to compare different methods to assess the arm stroke efficiency ($\eta_{F}$), when swimming front crawl using the arms only on the Measurement of Active Drag System (MAD System) and in a free-swimming condition, and to identify biophysical adaptations to swimming on the MAD System and the main biophysical predictors of maximal swimming speed in the 200 m front crawl using the arms only ($v_{200m}$). *Methods:* fourteen swimmers performed twice a 5 × 200 m incremental trial swimming the front crawl stroke using the arms only, once swimming freely, and once swimming on the MAD System. The total metabolic power was assessed in both conditions. The biomechanical parameters were obtained from video analysis and force data recorded on the MAD System. The $\eta_{F}$ was calculated using: (i) direct measures of mechanical and metabolic power (power-based method); (ii) forward speed/hand speed ratio (speed-based method), and (iii) the simplified paddle-wheel model. *Results:* both methods to assess $\eta_{F}$ on the MAD System differed (*p* < 0.001) from the expected values for this condition ($\eta_{F}$ = 1), with the speed-based method providing the closest values ($\eta_{F}$~0.96). In the free-swimming condition, the power-based ($\eta_{F}$~0.75), speed-based ($\eta_{F}$~0.62), and paddle-wheel ($\eta_{F}$~0.39) efficiencies were significantly different (*p* < 0.001). Although all methods provided values within the limits of agreement, the speed-based method provided the closest values to the “actual efficiency”. The main biophysical predictors of $v_{200m}$ were included in two models: biomechanical (R^2^ = 0.98) and physiological (R^2^ = 0.98). *Conclusions:* our results suggest that the speed-based method provides the closest values to the “actual $\eta_{F}$” and confirm that swimming performance depends on the balance of biomechanical and bioenergetic parameters

## 1. Introduction

The arm stroke efficiency in swimming has been usually represented by the fraction of the external mechanical power that is converted into useful propulsive power (i.e., Froude efficiency; $\eta_{F}$) and reported as one of the main determinants of swimming performance [1,2]. Thus, understanding and developing methods that are simultaneously reliable, feasible, and coach-friendly, should be a major concern in swimming research. Although several methods have been used to assess $\eta_{F}$ [3,4,5,6,7], it is unclear whether they provide accurate values and agree with each other. 

For instance, Toussaint et al. [3] suggested that $\eta_{F}$ could be obtained from a power-based method, in which direct assessments of the external mechanical power (${\dot{W}}_{ext}$) for a given metabolic power input, as well as the useful propulsive power (i.e., power to overcome drag; ${\dot{W}}_{d}$) for a given swimming speed, are extrapolated from a condition in which $\eta_{F}$ is forced to be maximal to a normal free-swimming condition. Considering the limitations imposed by the aquatic environment, Martin et al. [4] described a theoretical model of the arm stroke propulsion, from which $\eta_{F}$ could be obtained from a speed-based method that estimates the ratio of the average forward speed and the tangential hand speed (v), assuming propulsive and drag forces are the same for a given constant speed. This theoretical model was later adapted by Zamparo et al. [5] as a simplified paddle-wheel model to estimate $\eta_{F}$ during the underwater phase only, over half a cycle.

Considering that these methods might provide different values of $\eta_{F}$ for a given v, as indicated by the data reported in the literature, comparing them in a controlled condition in which no power is wasted to transfer kinetic energy to the water (${\dot{W}}_{k}=0)$, and hence $\eta_{F}$ is maximal, is the first step in identifying the potential differences and main limitations of each method. One way to impose a minimal ${\dot{W}}_{k}$, and a maximal $\eta_{F}$, has been the use of the system for measurement of active drag (MAD System), in which swimmers must push-off fixed pads to generate propulsion with no major changes in the swimming technique [3,8,9]. Identifying the biophysical adaptations to swimming on the MAD System, relative to free-swimming, could also reinforce theoretical assumptions on the interplay between swimming efficiency and economy, since experimental data are scarce and the anaerobic contribution is usually neglected [3,10,11,12].

Thus, the aims of this study were: (i) to compare the power-based, speed-based, and paddle-wheel methods to assess $\eta_{F}$ when swimming front crawl using the arms only, on the MAD System and in a free-swimming condition; (ii) to compare the biophysical responses to free-swimming and MAD System conditions, in a range of paired speeds, and (iii) to identify the main biophysical predictors of maximal swimming speed in the 200 m front crawl using the arms only ($v_{200m}$). We hypothesize that $\eta_{F}$ is underestimated when using the paddle-wheel model to calculate this parameter. Moreover, we expect that $v_{200m}$ is determined by the interplay between biomechanical and physiological parameters.

## 2. Materials and Methods

### 2.1. Participants

Fourteen national level competitive swimmers (eight males, six females) volunteered to this study (age: 17.3 ± 2.2 years; body mass: 65.3 ± 10.6 kg; height: 171.7 ± 9.9 cm). The purpose and the aims of the study were carefully explained to each individual, and written informed consent was obtained. The study conformed to the standards set by the Declaration of Helsinki, and the local Institutional Ethical Commission approved the procedures (No: 648.622).

### 2.2. Experimental Procedures

The experimental protocol consisted of two testing sessions separated by 8 h. During each session, swimmers completed a standardized warm-up followed by 5 × 200 m trials at pre-determined speeds (80%, 85%, 90%, 95%, and 100% of the maximal speed in free-swimming using the arms only). Testing took place in a 25 m, six lanes, 195 cm depth indoor swimming pool with a water temperature of 27.5 °C and a relative air humidity of 60%. All swimmers were familiarized and experienced with the apparatus used in the data collection.

The 5 × 200 m incremental trials were performed using the front crawl stroke with arms only, once swimming freely and once swimming on the MAD System. During each trial, v was controlled by a visual pacer with flashing lights at the bottom of the swimming pool (Pacer2Swim, KulzerTEC, Aveiro, Portugal). In both conditions, swimmers used a pull buoy and a rubber band around their ankles to avoid propulsion generated from the kick. In-water starts and open turns were used due to constraints imposed by the apparatus utilized for the physiological and biomechanical assessments. Passive recovery periods of at least 5 min were given to the participants after each step.

### 2.3. Physiological Assessments

Respiratory and pulmonary gas-exchange data were directly and continuously assessed breath-by-breath using a telemetric portable gas analyzer (K4b2, Cosmed, Rome, Italy) connected to a low hydrodynamic resistance respiratory snorkel and valve system (AquaTrainer, Cosmed, Rome, Italy) as reported by Ribeiro et al. [13]. The apparatus was suspended at 2 m above the water surface following the swimmer along the pool using a steel cable system designed to minimize disturbance of the normal swimming movements. The telemetric portable gas analyzer was calibrated before each test with gases of known concentration (16% oxygen and 5% carbon dioxide) and the turbine volume transducer calibrated with a 3 L syringe. Anomalous $\dot{V}O_{2}$ values greater than ±4 SD from the mean of the final 60 s of each step were manually removed before data were averaged. The average of the final 60 s of $\dot{V}O_{2}$ data (mL·kg^−1^·min^−1^) were used for analysis and calculations.

Capillary blood samples (5 µL) for lactate concentration ([La^−^]) analysis were collected from the earlobe at rest, at the end of each step and in the recovery periods (after 1, 3, and 5 min) and analyzed using a portable lactate analyzer (Lactate Pro 2, Arkay, Inc., Kyoto, Japan). The net [La^−^], in mmol·L^−1^, was then transformed into $\dot{V}O_{2}$ equivalents using a 2.7 mL·kg^−1^·mmol^−1^ constant [14,15]:

(1) $$\dot{V}O_{2}\left(An\right)=2.7\cdot {\left[La^{-}\right]}_{net}/t_{step}$$

where $\dot{V}O_{2}\left(An\right)$ represents the volume of oxygen (mL·kg^−1^·min^−1^) consumed over the duration of each step if the anaerobically produced energy had instead been produced via aerobic pathways and $t_{step}$ is the step duration (min).

Estimations of metabolic power produced by aerobic (${\dot{E}}_{aer}$) and anaerobic lactic pathways (${\dot{E}}_{aner}$) were converted to watts, considering the body mass of the swimmers and the energy equivalent of O_2_ (α), as previously described [16,17]:

(2) α=15.87+5.26·RER

(3) $${\dot{E}}_{aer}=\dot{V}O_{2}\cdot \alpha \cdot BM/60$$

(4) $${\dot{E}}_{aner}=\dot{V}O_{2}\left(An\right)\cdot \alpha \cdot BM/60$$

where RER is the respiratory exchange ratio and BM is the body mass, in kg.

The overall metabolic power input (${\dot{E}}_{tot}$) resulted from the sum of ${\dot{E}}_{aer}$ and ${\dot{E}}_{aner}$:

(5) $${\dot{E}}_{tot}={\dot{E}}_{aer}+{\dot{E}}_{anaer}$$

Finally, to obtain the energy cost of swimming (C, expressed in kJ·m^−1^), ${\dot{E}}_{tot}$ was converted to kJ·s^−1^ and divided by the swimming speed, as follows:

(6) $$C=\left({\dot{E}}_{tot}/1000\right)/v$$

### 2.4. Biomechanical Assessments in Free-Swimming

**Figure 1:**
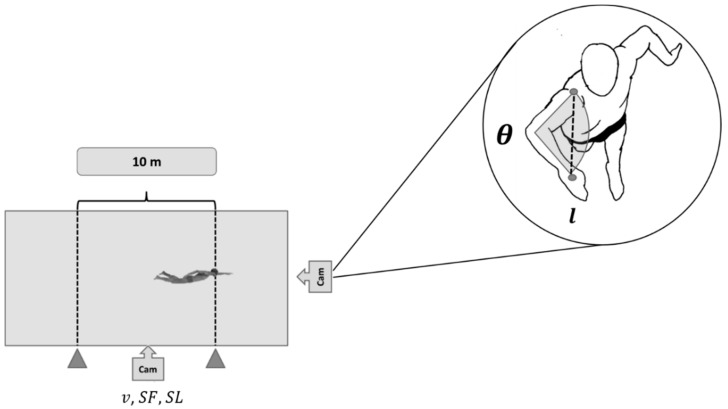
Stroke parameters assessed in the central 10 m of the swimming pool, as well as from a frontal camera recording the frontal plane of the swimmer. v: average swimming speed; *SF*: average stroke frequency; *SL*: average stroke length; *θ*: elbow angle at the end of the in-sweep phase; *l*: shoulder to hand distance.

Swimmers were recorded in the sagittal plane with a stationary video camera (50 Hz; HDR CX160E, Sony Electronics Inc., San Diego, CA, USA) positioned on the opposite side of the swimming pool. The space recorded was calibrated using lane marks measuring the central 10 m of the swimming pool (7.5 m to 17.5 m) (Figure 1). Video images were analyzed using a motion analysis software (Kinovea Version 0.8.15, Free Software Foundation, Boston, MA, USA) and the number of complete strokes recorded within the calibration marks and the time taken from the first and last entry of the same hand in the water was computed, yielding the average stroke frequency:

(7) $$SF_{free}=n_{strokes}/t_{strokes}$$

where $SF_{free}$ is the stroke frequency in the free-swimming condition, $n_{strokes}$ is the number of complete arm strokes, and $t_{strokes}$ is the time taken to complete them. The vertex was digitized in the same frames of the first and last hand entry in the water, allowing the calculation of the average swimming speed:

(8) $$v_{free}=d_{strokes}/t_{strokes}$$

in which $v_{free}$ is the actual swimming speed in the free-swimming condition, and $d_{strokes}$ is the distance covered by the vertex of the swimmer from the first and last hand entry of the same hand in the water. No differences higher than 0.01 m/s were observed between $v_{free}$ and the imposed swimming speed.

The average stroke length ($SL_{free}$) was calculated by combining Equations (7) and (8), as follows:

(9) $$SL_{free}=v_{free}/SF_{free}$$

An underwater video camera (50 Hz; HDR CX160E, Sony Electronics Inc., San Diego, CA, USA) positioned on the frontal wall in a waterproof case at 0.5 m below the water surface recorded the swimmer’s transverse plane perspective. The elbow angle was measured at the end of the in-sweep phase (when the plane of the arm and forearm is perpendicular to the optical axis of the camera) for the right and left sides and for, at least, six different arm strokes (three from each side). As shown in Figure 1, and described in Equation (10), the average elbow angle between both sides was then used to calculate shoulder to hand distance (l) by trigonometry considering the arm (from the lateral epicondyle of the humerus to the acromion process) and forearm lengths (from the center of the hand to the lateral epicondyle of the humerus) previously measured with a meter tape (0.01 cm resolution):

(10) $$l=\sqrt{l_{arm}{}^{2}+l_{forearm}{}^{2}-2\cdot l_{arm}\cdot l_{forearm}\cdot \cos\theta }$$

in which θ is the elbow angle in radians, $l_{arm}$ and $l_{forearm}$ are the arm and forearm lengths in m, respectively. 

### 2.5. Biomechanical Assessments on the MAD System

**Figure 2:**
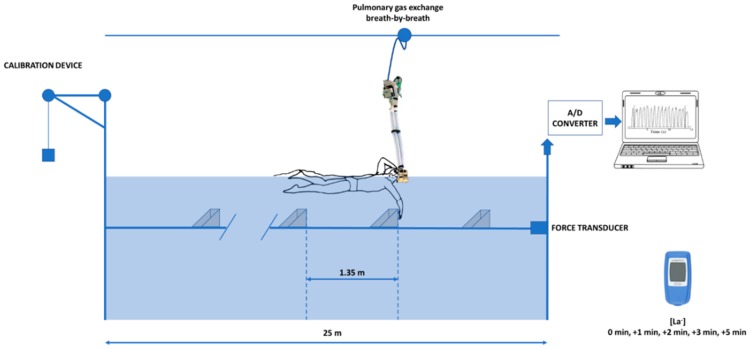
The structure of the Measurement of Active Drag (MAD) System. Forces were applied on the push-off pads and assessed for each arm stroke by a force transducer.

When swimming on the MAD System, propulsion was generated without wasting kinetic energy to the water (${\dot{W}}_{k}=0$), and therefore, $\eta_{F}=1$ [3]. Swimmers pushed-off from fixed pads attached to a 23 m rod placed 0.8 m below the water surface, with l fixed at 0.45 m, and with a standard inter-pad distance of 1.35 m (16 pads in total). The rod is instrumented with a force transducer, allowing the measurement of a direct push-force at each pad and the calculation of the mean force at each lap, as presented in Figure 2.

The force signal was acquired using an A/D converter (BIOPAC Systems, Inc.) at a sample rate of 1000 Hz and filtered with a low-pass digital filter with a cut-off frequency of 10 Hz [13]. The first and last push-off were neglected to eliminate the influence of the push-off from the wall (first pad) and the deceleration of the swimmer at the end of the lane (last pad). The remaining force signal was time-integrated, yielding the average force at each lap. 

The actual swimming speed was computed from the force signal, considering the time needed to cover the distance between the second and the last pad (18.9 m), and no differences larger than 0.01 m/s from the imposed swimming speed were observed. The average stroke frequency in this condition ($SF_{MAD}$) was calculated from the imposed swimming speed and the inter-pad distance ($d_{inter-pad}$), as follows:

(11) $$SF_{MAD}=v/2\cdot d_{inter-pad}$$

Assuming each swimmer performed at a constant swimming speed, their mean exerted force was equal to the mean drag force, with the five velocity/drag ratio data being least square fitted in a power function, as follows:

(12) $$D=k\cdot v^{n}$$

in which D is the total active drag, v is the average swimming speed and k (speed-specific drag) and n are parameters of the power function. The power to overcome drag (${\dot{W}}_{d}$) was calculated as the product of v and the correspondent D:

(13) $${\dot{W}}_{d}=D\cdot v$$

The power needed to overcome the external forces (${\dot{W}}_{ext}$) is determined by:

(14) $${\dot{W}}_{ext}=F_{hand}\cdot v_{hand}$$

in which $F_{hand}$ is the resultant propulsive force exerted by the hand and $v_{hand}$ is the average effective hand speed. 

The ${\dot{W}}_{ext}$ can be partitioned in the power needed to overcome drag forces (${\dot{W}}_{d}$) and the power needed to give the water kinetic energy (${\dot{W}}_{k}$):

(15) $${\dot{W}}_{ext}={\dot{W}}_{d}+{\dot{W}}_{k}$$

Since no power is wasted to the water when swimming on the MAD System (${\dot{W}}_{k}=0$), ${\dot{W}}_{d}$ was equal to the external mechanical power output (${\dot{W}}_{ext}$) in this condition:

(16) $${\dot{W}}_{ext}={\dot{W}}_{d}$$

### 2.6. Speed-Based Efficiency

The speed-based $\eta_{F}$ was assessed in the MAD System and in free-swimming conditions by combining Equations (13) and (14), yielding:

(17) $$\eta_{F}=\left(D\cdot v\right)/\left(F_{hand}\cdot v_{hand}\right)$$

in which $F_{hand}$ is assumed to be equal to D for a given constant speed, and $v_{hand}$ is calculated with a model proposed by Martin et al. [4]. In this model, the arm is considered a rigid segment (l) rotating at constant angular speed (ω) around the shoulder:

(18) $$v_{hand}=\omega \cdot l$$

The average ω was estimated based on the ratio of the circumference traveled by the hand in the model and its diameter (π ≈ 3.14) and SF values:

(19) ω=2π·SF

Thus, $\eta_{F}$ can be calculated as follows:

(20) $$\eta_{F}=v/v_{hand}$$

### 2.7. Paddle-Wheel Efficiency

The “paddle-wheel” arm stroke (Froude) efficiency was calculated according to the model proposed by Zamparo et al. [5], adapted from Martin et al. [4], that yields the theoretical efficiency of the underwater phase only, as follows:

(21) $$\eta_{F}=v/(v_{hand}\cdot 2/\pi )$$

### 2.8. Power-Based Efficiency

At each step, a mean value of ${\dot{W}}_{ext}$ was calculated from the eight lengths swam over the MAD System, thus the linear relationship between ${\dot{E}}_{tot}$ and ${\dot{W}}_{ext}$ was obtained, and the individual regression equations were used to calculate ${\dot{E}}_{tot}$ in free-swimming. Since ${\dot{W}}_{d}$ was known for each swimmer in each speed from the measurements on the MAD System, Froude efficiency in free-swimming could be calculated:

(22) $$\eta_{F}={\dot{W}}_{d}/{\dot{W}}_{ext}$$

where $\eta_{F}$ is the Froude efficiency, which represents the fraction of the ${\dot{W}}_{ext}$ that is converted into useful propulsive power (${\dot{W}}_{d}$).

### 2.9. Statistical Analysis

Descriptive statistics are reported for all variables (mean ± SD). The normality of the data distribution was tested with a Shapiro–Wilk’s test, and Levene’s test was applied to verify the equality of the variances. Mauchly’s sphericity test was used to validate the subsequent comparison tests. A two-way repeated-measures ANOVA was applied for the data comparison regarding the effects of the method and of the swimming speed on the arm stroke efficiency parameters. When any significant effect was identified, Bonferroni’s posthoc analysis was performed to compare the different paces, conditions, or methods. If an interaction between factors occurred, the simple effect of each factor on each level of the other factor was calculated. Effect sizes were estimated using the partial η^2^ to describe the proportion of the total variance made up by the variance of the means. The ratio of variance explained of the sample was calculated for each effect and parameter estimate. Interpretation of η^2^ indicates small (η^2^ ≥ 0.02), medium (η^2^ ≥ 0.13), or large effect sizes (η^2^ ≥ 0.26) for a two-way ANOVA and small (η^2^ ≥ 0.01), medium (η^2^ ≥ 0.06), or large effect sizes (η^2^ ≥ 0.14) for a one-way ANOVA according to the general rules of thumb on magnitudes of effect sizes [18]. In addition, Bland–Altman plots [19] were used to establish an agreement between the $\eta_{F}$ estimated from the different methods.

To identify the main predictors of $v_{200m}$, a principal component analysis was performed to convert the set of observations of possibly correlated variables into a set of values of linearly uncorrelated variables, reducing the number of dimensions. The two main principal components were considered for the analysis, and the variables that presented loading values ≥0.8 were selected for multiple linear regression, excluding the redundant variables from the model.

For all analyses, the level of significance adopted was *p* ≤ 0.05.

## 3. Results

**Figure 3:**
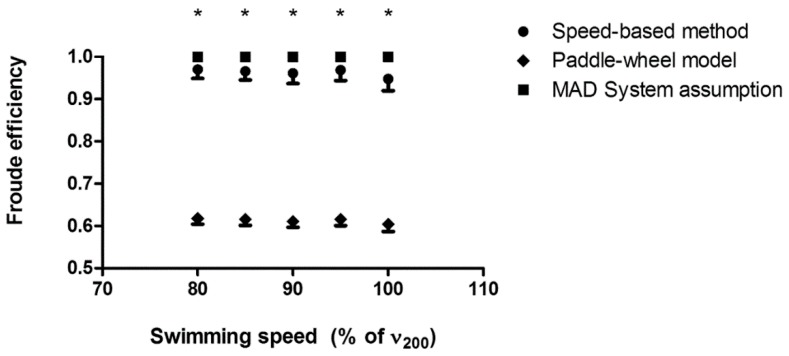
Values of stroke efficiency assessed in the MAD System condition by different methods in a range of speeds, from 80 to 100% of $v_{200}$; * All methods were different for each swimming speed (*p* < 0.001).

No effects of swimming speed on the arm stroke efficiency were observed in the MAD System condition (*p* > 0.05). The average difference between the speed-based and the theoretical efficiency assumed for the MAD System was 0.04 ± 0.02 (~4%; *p* < 0.001). The difference between the paddle-wheel efficiency and the theoretical assumption for the MAD System was 0.39 ± 0.02 (~39%; *p* < 0.001). When comparing the paddle-wheel model and the speed-based method, values of arm stroke efficiency were, in average, 0.35 ± 0.01 higher in the latter (~35%; *p* < 0.001). The individual values of arm stroke efficiency for each speed is presented in Figure 3.

**Figure 4:**
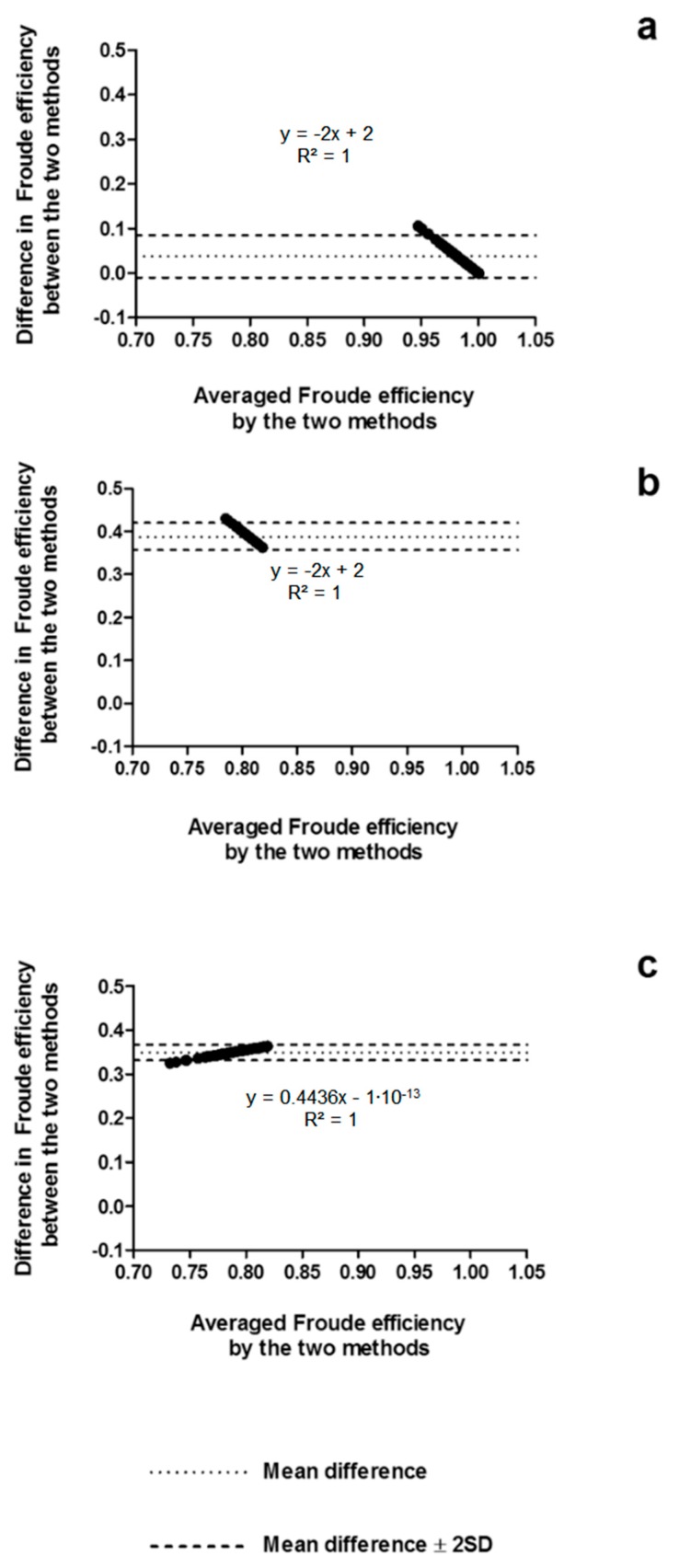
Bland–Altman plots testing the agreement between the speed-based efficiency and the MAD System assumption (**a**), paddle-wheel and MAD System assumption (**b**), and paddle-wheel and speed-based efficiency (**c**). SD: standard deviation.

The agreement between methods is presented in Figure 4, indicating a short amplitude of the limits of agreement when comparing the speed-based method and the MAD System assumption (between −0.01 and 0.08), the paddle-wheel model and the MAD System assumption (between 0.33 and 0.37), and the paddle-wheel model and the speed-based method (between 0.36 and 0.42). Moreover, the differences seemed to be influenced by the magnitude of the averaged efficiency between the methods (R^2^ = 1; *p* < 0.001), as indicated in the linear regression equations of each Bland–Altman plot.

**Figure 5:**
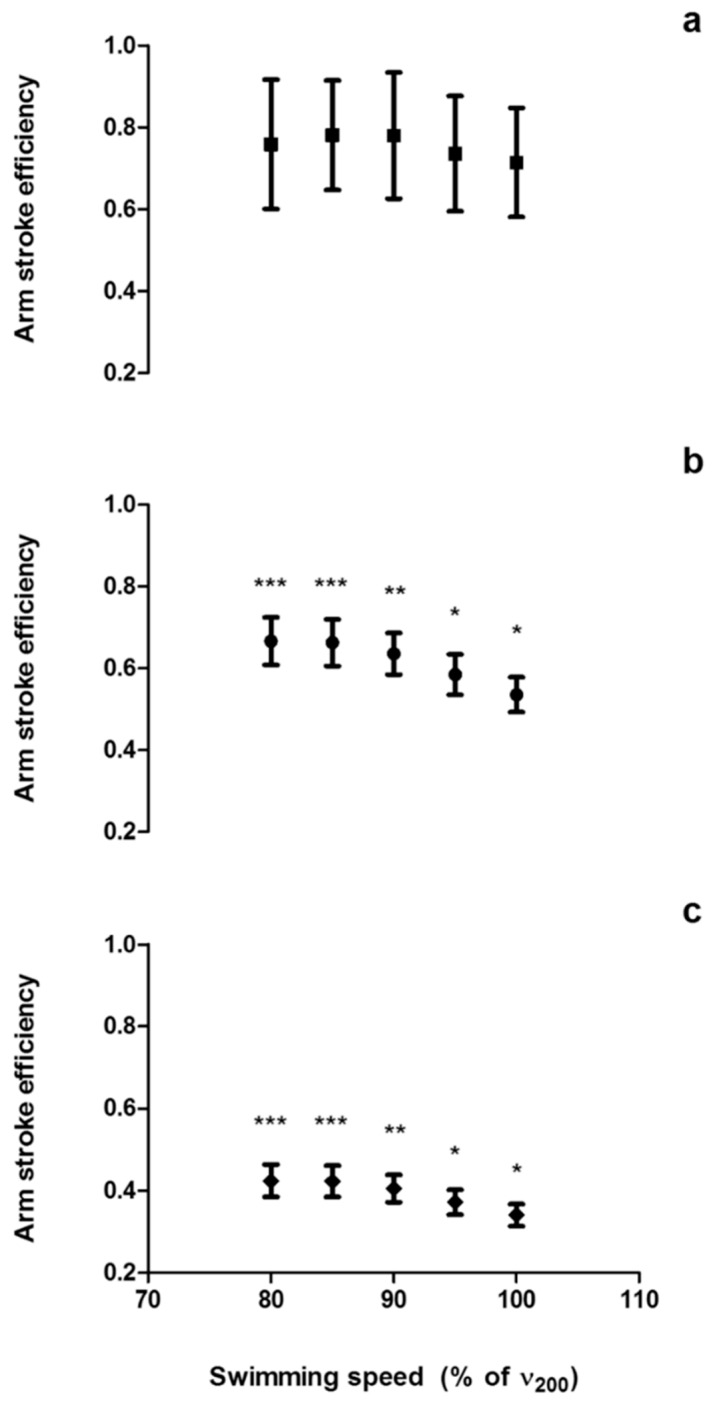
Froude efficiency assessed by the power-based (**a**), paddle-wheel (**b**), and speed-based (**c**) methods at different speeds, during the incremental protocol; *** Different from arm stroke efficiency values at 95 and 100% of $v_{200m}$ (*p* < 0.05); ** Different from arm stroke efficiency values at 85%, 95%, and 100% of $v_{200m}$ (*p* < 0.05); * Different from arm stroke efficiency values at all swimming speeds (*p* < 0.05).

In free-swimming, there was an interaction between swimming speed and method to assess the arm stroke efficiency (*p* = 0.025). No differences were found in power-based efficiency between the different speeds (*p* > 0.05). The arm stroke efficiency assessed using the speed-based and paddle-wheel methods significantly decreased from 80 to 100% of $v_{200}$ (*p* < 0.001). The individual comparisons of the arm stroke efficiency between the different speeds for each method are presented in Figure 5.

**Figure 6:**
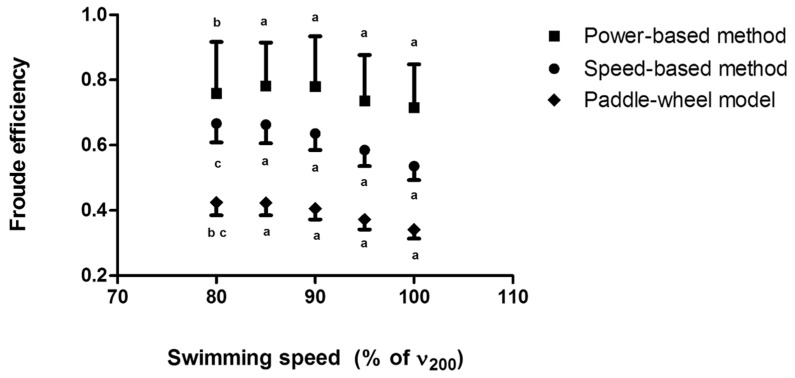
Froude efficiency assessed by the power-based, paddle-wheel, and speed-based methods at different speeds during the incremental protocol; a. All methods are different; b. Difference between the power-based method and the paddle-wheel model; c. Difference between the speed-based method and the paddle-wheel model.

The individual comparisons of the arm stroke efficiency between the different methods are presented in Figure 6.

**Figure 7:**
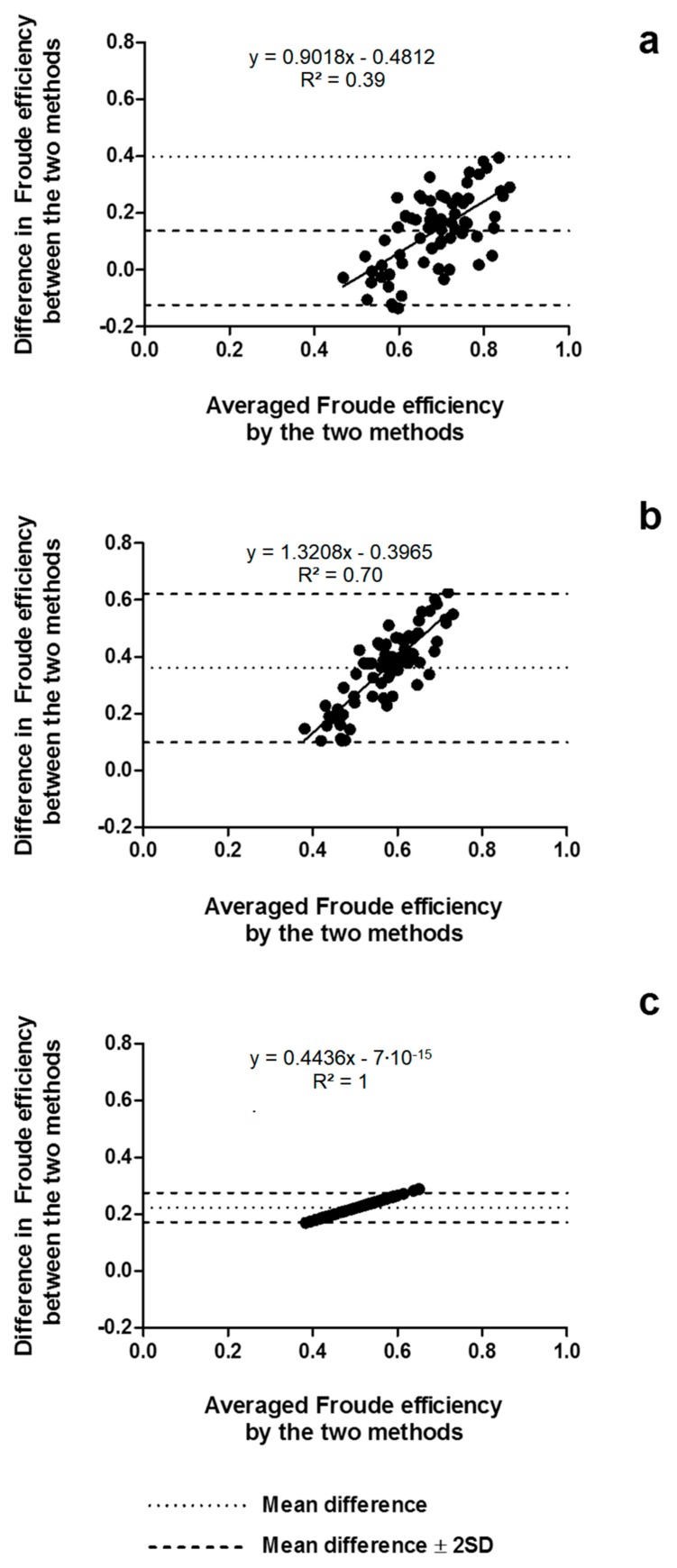
Bland–Altman plots testing the agreement between the speed-based and power-based efficiencies (**a**), paddle-wheel and power-based efficiency (**b**), and paddle-wheel and speed-based efficiency, and (**c**) in the free-swimming condition.

In this condition, the speed-based method provided ~16% lower efficiency than the power-based method (average difference: −0.14 ± 0.13; *p* < 0.001), the paddle-wheel efficiency was ~46% lower than the power-based method (average difference: −0.36 ± 0.13; *p* < 0.001), and ~36% lower than the speed-based method (average difference: −0.22 ± 0.03; *p* < 0.001). The differences between the methods were within the limits of agreement and seemed to be determined by the magnitude of the averaged arm stroke efficiency between methods, as shown in Figure 7.

All swimming speeds were different from each other (*p* < 0.001), as expected. Significant effects of swimming speed on D, SF, and SL were observed (*p* < 0.001). Also, the swimming condition had a significant effect on SF and SL (*p* < 0.001). Moreover, an interaction between the swimming speed and swimming condition was observed for SF and SL (*p* < 0.001). 

Values of ${\dot{W}}_{ext}$, ${\dot{W}}_{d}$, and ${\dot{W}}_{k}$ increased with swimming speed (*p* < 0.001). In addition, ${\dot{W}}_{ext}$ and ${\dot{W}}_{k}$ decreased in the MAD System condition in comparison to free-swimming (*p* < 0.001). The interaction between swimming speed and swimming condition for ${\dot{W}}_{ext}\;$(*p* < 0.001) and ${\dot{W}}_{k}$ (*p* < 0.001) made it possible to compare these parameters individually between the different steps and the different swimming conditions. 

The mean (±SD) values of the biomechanical parameters, as well as the individual differences between each step and between free-swimming and MAD System conditions are reported in Table 1.

**Figure 8:**
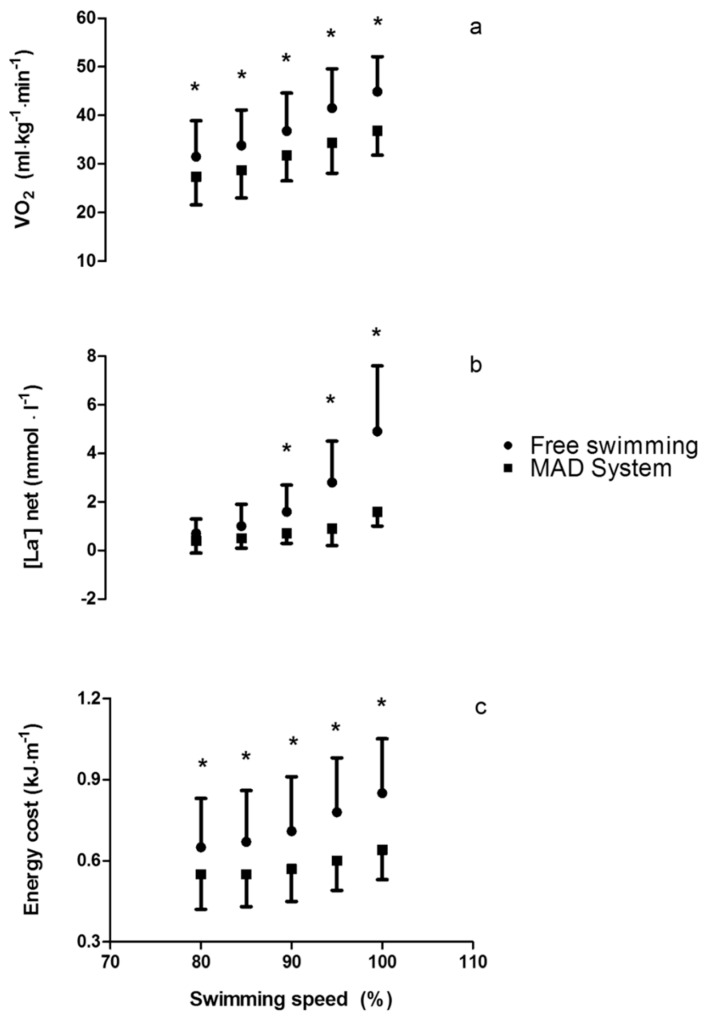
Individual differences in oxygen uptake (**a**), blood lactate concentration, (**b**) and energy cost (**c**) between free-swimming and the MAD System condition for each imposed speed (* *p* < 0.05).

Significant effects of swimming speed were observed for metabolic parameters, indicating that $\dot{V}O_{2}$, [La^−^]_net_, and C increase with speed (*p* < 0.001). Moreover, swimming on the MAD System promoted a reduction in $\dot{V}O_{2}$ (*p* < 0.001), [La^−^]_net_ (*p* = 0.001), and C (*p* < 0.001) for equivalent speeds. The interaction between swimming speed and swimming condition allowed the individual comparisons between each step and each condition for the $\dot{V}O_{2}$ (*p* = 0.006), [La^−^]_net_ (*p* < 0.001) and C (*p* < 0.001). The mean (±SD) values of the metabolic parameters, as well as the individual differences between the free-swimming and MAD System conditions, are presented in Figure 8. Values of $\dot{V}O_{2}$ ranged from 31.5 ± 7.4 to 44.9 ± 7.2 mL·kg^−1^·min^−1^ in free-swimming and from 27.4 ± 5.8 to 36.8 ± 5.0 mL·kg^−1^·min^−1^ in the MAD System condition; [La^−1^]_net_ ranged from 0.7 ± 0.5 to 4.9 ± 2.7 mmol·L^−1^ in free-swimming and from 0.4 ± 0.5 to 1.6 ± 0.6 mmol·L^−1^ in the MAD System condition; and C ranged from 0.65 ± 0.18 to 0.85 ± 0.20 kj·m^−1^ in free-swimming and from 0.55 ± 0.13 to 0.64 ± 0.11 kj·m^−1^ in the MAD System condition.

Swimming speed had a significant effect on ${\dot{E}}_{aer}$, ${\dot{E}}_{aner}$, ${\dot{E}}_{tot}$, aerobic and anaerobic contributions (*p* < 0.001). Significant effects of swimming condition on ${\dot{E}}_{aer}$ (*p* < 0.001), ${\dot{E}}_{aner}$ (*p* = 0.002), ${\dot{E}}_{tot}$ (*p* < 0.001), aerobic contribution (*p* < 0.001), and anaerobic contribution (*p* < 0.001) were also observed. Interaction between swimming speed and condition for ${\dot{E}}_{aer}$ (*p* = 0.001), ${\dot{E}}_{aner}$ (*p* < 0.001), ${\dot{E}}_{tot}$(*p* < 0.001), aerobic contribution (*p* < 0.001), and anaerobic contribution (*p* < 0.001), allowed the individual comparisons between each step of the protocol and between each swimming condition, as presented in Table 2.

Among the variables selected from the principal component analysis, the redundant parameters were excluded. The loading values of each variable in the first two principal components are presented in Table 3.

The selected parameters were divided into biomechanical ($\eta_{F}$, ${\dot{W}}_{ext}$, and k) and physiological (${\dot{E}}_{tot}$ and C) prediction models. The multiple linear regressions indicated that all the parameters were significant determinants of the prediction models (*p* < 0.001). Both biomechanical (R^2^ = 0.98; *p* < 0.001) and physiological (R^2^ = 0.98; *p* < 0.001) models could significantly predict the variances in $v_{200}$ and are presented in Equations (23) and (24):

(23) $$v_{200}=0.003\cdot {\dot{W}}_{ext}+0.754\cdot \eta_{F}-0.012\cdot k+0.0873$$

(24) $$v_{200}=0.001\cdot {\dot{E}}_{tot}-1.643\cdot C+1.315$$

## 4. Discussion

This study aimed to compare the different available methods to assess the arm stroke efficiency in front crawl swimming with the arms only, in two conditions: swimming on the MAD System and free-swimming. The main biophysical effects of swimming on the MAD System were identified, and two prediction models were established to explain the variances in $v_{200}$.

### 4.1. Arm Stroke Efficiency in the MAD System and Free-Swimming Conditions

Although the three ways to estimate $\eta_{F}$ when swimming on the MAD System were significantly different, our results indicate that the speed-based method provides the closest values to the theoretical arm stroke efficiency for this condition ($\eta_{F}=1$), in which the power waisted in transferring kinetic energy to the water is neglected, assuming swimming speed is constant [3]. Values of speed-based $\eta_{F}$ ranged from 0.9 to 1 and were, on average, ~4% lower than the theoretical $\eta_{F}$ expected for the MAD System. This method was first reported by Martin et al. [4] as a model to describe the hand propulsion in front crawl swimming, in which the arm is considered a rigid segment of length l, rotating at constant angular speed around the shoulder. The main assumption of this method is that the active drag and the effective force applied by the hand are the same for a given constant speed. Therefore, $\eta_{F}$ results from the ratio of the tangential hand speed and the average forward speed, as described in Equation (20). This approach has been adapted by Zamparo et al. [5] as a simplified paddle-wheel model, with the purpose of calculating the arm stroke efficiency during the underwater phase only, over half a stroke cycle. Although kinematical models of the arm stroke propulsion have been largely used to assess the arm stroke efficiency in front crawl swimming [2,4,5,20,21,22], to our knowledge, this is the first study comparing these methods to the theoretical efficiency when swimming on the MAD System.

The outcomes of the simplified paddle-wheel model were significantly lower than those of the theoretical efficiency when swimming on the MAD System (~39%) and of the speed-based method in this condition (~35%). The magnitude of the differences between the paddle-wheel and speed-based values was nearly the same in the free-swimming condition (~36%). Both speed-based and paddle-wheel methods assume that propulsion is generated by a rigid segment rotating at a constant speed around the shoulder [4,5]. The conceptual difference between these methods is that the paddle-wheel model includes a component to the equation initially proposed by Martin et al. [4], aiming to consider only the underwater phases of the arm stroke over half a cycle (i.e., a single-arm stroke), from 0 to π [5]. However, the adaptation proposed by Zamparo et al. [5] seems to be conflictual with the original assumptions of the model. By assuming the arm is rotating at a constant angular speed around the shoulder, the method considers that the average angular speed of the propelling segment is the same in the aerial and underwater phases of the arm stroke and that there is not an overlap between propulsive actions generated by each upper-limb. Therefore, the initial equation proposed by Martin et al. [4], in which $\eta_{F}$ is based on the ratio of $v_{hand}$ (calculated from SF values) and v (Equation (20)), should not be adjusted for this purpose. In fact, the duration of the underwater and aerial phases of the arm stroke is not necessarily the same [23], and the calculation of the arm stroke efficiency is meaningful for the propulsive phase only. Thus, although differences between the speed-based method and the theoretical efficiency assumed for the MAD System condition were small, they were possibly related to eventual propulsive gaps between pads. The only way to avoid such miscalculations of the $\eta_{F}$ would be considering $v_{hand}$ and v during the propulsive phases only, using the original model proposed by Martin et al. [4].

In addition, in the recent study of Gatta, Cortesi, Swaine, and Zamparo [24], the authors attempted to “validate” the paddle-wheel model by comparing: (i) values of arm stroke efficiency obtained with this method and (ii) the ratio between propulsive power and external mechanical power. To this aim, propulsive power was estimated by the product of the mean tethered force in a 15 s tethered swimming test and the maximal swimming speed in 25 m, whilst the “total mechanical power” was estimated in a whole-body swimming ergometer test, considering the power exerted by the upper limbs as well as of the lower limbs as the sum of the power assessed by the left and right sides, as previously described by Zamparo and Swaine [25]. However, this leads to an overestimation of “total mechanical power”, since the front crawl is a technique in which propulsion is generated by each limb (left or right) alternately, regardless of the coordination pattern. Thus, the correct way to calculate the mechanical power of the arms and legs should be to average the mechanical power output of the left and right limbs. Therefore, the actual values of mechanical power in the whole-body swimming ergometer test should be nearly half of the values presented in their study (~470 W instead of ~940 W) and, consequently, the values of “propelling efficiency” calculated as the ratio of thrust power and “total mechanical power” should be nearly twice the values presented in their study (~0.80 instead of ~0.40) and twice the values of the efficiency obtained with the paddle-wheel model (~0.80 vs. ~0.40).

Differently than in the MAD System condition, in which lower differences were found between the speed-based efficiency and the theoretical efficiency assumed for that condition, no “real” efficiency could be used to compare methods in free-swimming. Relatively to the power-based method, a larger difference in the speed-based (~16%) and paddle-wheel values (~46%) was observed, which could be caused, at least partially, by a longer duration of non-propulsive phases in this condition, since swimmers were not constrained to generate propulsion by pushing-off fixed points. The higher values of power-based efficiency could also be related to the several assumptions of this method [3], especially for considering ${\dot{E}}_{tot}$ as the only predictor of ${\dot{W}}_{ext}$, which may lead to a miscalculation.

It should be highlighted, however, that despite that all the methods provided significantly different values of $\eta_{F}$, they agreed with each other, and they might be considered as valid methods to measure efficiency, although not interchangeably, as indicated in Figure 4 and Figure 7. The amplitude of the limits of agreement was shorter in the MAD System condition, which is possibly related to the “fixed” values of $\eta_{F}$ assumed for this condition, reducing the variability in the averaged efficiency and in the differences between the methods. Furthermore, since speed-based and paddle-wheel $\eta_{F}$ were obtained from the same parameters, the variances in $\eta_{F}$ obtained from both methods are similar, resulting in shorter limits of agreement when comparing these methods, in both conditions. Especially in the free-swimming condition, the linear regressions provided by the agreement analysis indicated that differences between methods are determined by the magnitude of the averaged efficiency, which means that differences are higher at high-efficiency values (and low swimming speeds) and closer to 0 at lower efficiency values (and higher swimming speeds).

### 4.2. Biophysical Adaptations to Enhance Efficiency

When swimming on the MAD system, the arm stroke efficiency was enhanced, since it was forced to “maximal” [3]. Assuming ${\dot{W}}_{d}$ is the same in both conditions for a given constant speed, when swimmers are submitted to a condition in which the arm stroke efficiency is reduced, as is the case for free-swimming, the ${\dot{W}}_{k}$, and ${\dot{W}}_{ext}$ will be higher (see Equations (15), (16), and (22)). In fact, our results indicate a reduction of ~34% in ${\dot{W}}_{ext}$ at the lowest swimming speeds and of ~47% at $v_{200}$ when swimming on the MAD System, relative to free-swimming at paired swimming speeds, which is in accordance with data reported in previous studies for low submaximal speeds [1,3,10]. The reduced values of ${\dot{W}}_{k}$ and ${\dot{W}}_{ext}$ when swimming on the MAD System lead to a reduction in SF (18–33%) and an increase in SL (22–51%). These results confirm that the arm stroke efficiency is directly related to the SL and inversely related to the SF, as previously reported by Toussaint et al. [3] and Zamparo et al. [5].

The biomechanical adaptations to the MAD System condition were followed by an increase in the swimming economy. When forcing swimmers to perform at “maximal” arm stroke efficiency, the energy cost, as well as the ${\dot{E}}_{tot}$, reduced significantly (~16–24% in the range of speeds studied). Such adaptations have been previously reported by Toussaint et al. [3], although they neglected the anaerobic contribution by submitting swimmers to low submaximal intensities only. Our results indicate that the anaerobic contribution to the total metabolic power is not negligible, and increases with the swimming speed, as previously reported [16,26]. Moreover, the adaptions to the MAD System condition have shown that the anaerobic contribution reduces when increasing the arm stroke efficiency at a given swimming speed, suggesting that swimmers could sustain a given speed for a longer duration when enhancing efficiency.

Overall, our findings suggest that swimming on the MAD System might be a useful approach to increase the useful components of the mechanical power for a given metabolic demand, or even increasing the maximal power output, as suggested by Toussaint and Vervoorn [12]. Increasing the propelling surface area could also be used for this purpose, as reported by Toussaint et al. [1], although the long-term biophysical adaptations to training in these conditions are still unclear. 

### 4.3. Biophysical Predictors of Maximal Swimming Speed

The biomechanical prediction model was composed of $\eta_{F}$, ${\dot{W}}_{ext}$, and k, explaining 98% of the variances in $v_{200m}$. Therefore, the highest speeds can be achieved by the combination of high values of $\eta_{F}$ and ${\dot{W}}_{ext}$ (hence high ${\dot{W}}_{d}$ and low ${\dot{W}}_{k}$), accompanied by low values of k (related to the hydrodynamic resistance), supporting the theoretical relationship provided from the combination of Equations (12) and (13) [27,28]:

(25) $${\dot{W}}_{d}\approx k\cdot v³$$

Thus, by combining Equations (22) and (25), the relationship between the biomechanical predictors and swimming speed is determined:

(26) $$v³\approx ({\dot{W}}_{ext}\cdot \eta_{F})/k$$

A similar prediction model was reported by Zamparo et al. [2], in which ~75% of the variability in $v_{200m}$ could be explained by the variability in $\eta_{F}$ and ${\dot{W}}_{ext}$ (assessed with an arm crank ergometer). Relatively to the prediction model reported by Zamparo et al. [2], the quality of our prediction has increased by considering k, which is, in fact, another source of variability in maximal swimming speed [2,29]. Another reason that could explain the higher quality of our prediction might be related to the method used to assess the ${\dot{W}}_{ext}$, since in our study, ${\dot{W}}_{ext}$ was based on actual front crawl swimming assessments instead of a dryland protocol.

Two main physiological predictors were identified from the principal components analysis (C and ${\dot{E}}_{tot}$) and included in a regression that explained 98% of the variability in $v_{200m}$. The interplay between C and ${\dot{E}}_{tot}$ in determining maximal swimming performance is described in Equation (5), in which v is directly related to the capability of producing a high ${\dot{E}}_{tot}$, and inversely related to C, supporting the theoretical basis of the limiting factors of swimming performance [16,17,30]. The two prediction models defined in our study are not independent of each other, even though they could explain the variability in $v_{200m}$ individually. Swimming performance depends, in fact, on the interplay between biomechanical and bioenergetic parameters [31,32,33,34]. For instance, an increase in $\eta_{F}$ will always be accompanied by a reduction in C for a given swimming speed [5,35]. Likewise, any increase in the ${\dot{E}}_{tot}$ will allow a swimmer to produce a higher ${\dot{W}}_{ext}$ [3,25].

## 5. Conclusions

Although methods to assess the arm stroke efficiency on the MAD System differed from the expected values for this condition ($\eta_{F}=1)$, the speed-based method provided the closest values ($\eta_{F}\sim 0.96)$. The small difference between the MAD System assumption and the speed-based efficiency might be related to the assumptions of this method, that does not distinguish the propulsive and non-propulsive phases of the arm stroke. The large differences between the paddle-wheel assumption and the other methods may indicate that the way this method attempts to distinguish the underwater and aerial phases of the arm stroke is inadequate. In free-swimming, all methods (power-based, speed-based, and paddle-wheel model) provided different values of arm stroke efficiency, although values were within the limits of agreement of the Bland–Altman plots.

The arm stroke efficiency was enhanced in the MAD System condition, relatively to free-swimming, which lead to mechanical adaptations that included a reduction in stroke frequency and an increase in stroke length, reducing the external mechanical power output in a range of paired swimming speeds, from 80% to 100% of $v_{200m}$. These effects were followed by metabolic adaptations, with a decrease in energy cost and total metabolic power input for a given speed. Moreover, $\eta_{F}$, ${\dot{W}}_{ext}$, and k (biomechanical prediction model), as well as C and ${\dot{E}}_{tot}$ (physiological prediction model), were the main determinants of $v_{200m}$, confirming that swimming performance depends on the balance of biomechanical and bioenergetic parameters. 

## Figures and Tables

**Table 1:** Biomechanical parameters in free-swimming and Measurement of Active Drag (MAD) System conditions. Values of ${\dot{W}}_{ext}$ and ${\dot{W}}_{k}$ in free-swimming were obtained from the speed-based method to assess the arm stroke efficiency.

Step	Swimming Speed (%$v_{200}$ and m·s^−1^)		Active Drag (N)	Speed-Specific Drag	Stroke Frequency (Hz)		Stroke Length (m)		${\dot{W}}_{ext}$ (W)		${\dot{W}}_{d}$ (W)		${\dot{W}}_{k}$ (W)	
	Both Conditions		Both Conditions	Both Conditions	Free-Swimming	MAD System	Free-Swimming	MAD System	Free-Swimming	MAD System	Free-Swimming	MAD System	Free-Swimming	MAD System
1	80%	1.09 ± 0.09 ^b^	43.0 ± 11.1 ^b^	36.6 ± 9.4	0.49 ± 0.04 ^a,b^	0.40 ± 0.03 ^a,b^	2.22 ± 0.23 ^a c^	2.70 ± 0.00 ^a^	72 ± 23 ^a,b^	47 ± 14 ^a,b^	47 ± 14 ^b^	47 ± 14 ^b^	25 ± 11 ^a,b^	0 ± 0 ^a^
2	85%	1.15 ± 0.09 ^b^	47.7 ± 11.7 ^b^	35.9 ± 8.3	0.53 ± 0.04 ^a,b^	0.42 ± 0.04 ^a,b^	2.20 ± 0.17 ^a c^	2.70 ± 0.00 ^a^	85 ± 28 ^a,b^	55 ± 16 ^a,b^	55 ± 16 ^b^	55 ± 16 ^b^	30 ± 13 ^a,b^	0 ± 0 ^a^
3	90%	1.22 ± 0.10 ^b^	52.6 ± 12.3 ^b^	35.4 ± 7.5	0.58 ± 0.06 ^a,b^	0.45 ± 0.04 ^a,b^	2.12 ± 0.15 ^a c^	2.70 ± 0.00 ^a^	104 ± 33 ^a,b^	65 ± 18 ^a,b^	65 ± 18 ^b^	65 ± 18 ^b^	39 ± 16 ^a,b^	0 ± 0 ^a^
4	95%	1.29 ± 0.10 ^b^	57.7 ± 13.3 ^b^	34.8 ± 6.7	0.65 ± 0.07 ^a,b^	0.47 ± 0.04 ^a,b^	1.97 ± 0.14 ^a,b^	2.70 ± 0.00 ^a^	130 ± 42 ^a,b^	75 ± 21 ^a,b^	75 ± 21 ^b^	75 ± 21 ^b^	55 ± 22 ^a,b^	0 ± 0 ^a^
5	100%	1.35 ± 0.10 ^b^	63.3 ± 14.4 ^b^	34.4 ± 6.2	0.76 ± 0.08 ^a,b^	0.51 ± 0.04 ^a,b^	1.79 ± 0.11 ^a,b^	2.70 ± 0.00 ^a^	165 ± 52 ^a,b^	87 ± 24 ^a,b^	87 ± 24 ^b^	87 ± 24 ^b^	78 ± 29 ^a,b^	0 ± 0 ^a^

^a^ Different from the other condition (*p* < 0.05); ^b^ Different from all steps (*p* < 0.05).

**Table 2:** Partitioning of the metabolic power input in free-swimming and MAD System conditions.

Step	${\dot{E}}_{aer}$ (W)		${\dot{E}}_{anaer}$ (W)		${\dot{E}}_{tot}$ (W)		Aerobic Contribution (%)		Anaerobic Contribution (%)	
	Free Swimming	MAD System	Free Swimming	MAD System	Free Swimming	MAD System	Free Swimming	MAD System	Free Swimming	MAD System
1	702 ± 222 ^a c^	598 ± 166 ^a c^	15 ± 15 ^c^	9 ± 10 ^e^	716 ± 230 ^a c^	600 ± 173 ^a b^	98 ± 1 ^c^	99 ± 1 ^d^	2 ± 1 ^c^	1 ± 1 ^d^
2	759 ± 241 ^a c^	629 ± 171 ^a c^	23 ± 25 ^c^	10 ± 10 ^e^	782 ± 255 ^a c^	648 ± 181 ^a b^	97 ± 2 ^c^	99 ± 1 ^d^	3 ± 2 ^c^	1 ± 1 ^d^
3	833 ± 260 ^a b^	699 ± 160 ^a b^	39 ± 31 ^a b^	16 ± 11 ^a d^	873 ± 283 ^a b^	707 ± 186 ^a b^	96 ± 2 ^a b^	98 ± 1 ^a d^	4 ± 2 ^a b^	2 ± 1 ^d^
4	948 ± 276 ^a b^	763 ± 186 ^a b^	71 ± 52 ^a b^	23 ± 21 ^a d^	1018 ± 316 ^a b^	784 ± 192 ^a b^	94 ± 3 ^a b^	97 ± 2 ^a d^	6 ± 3 ^a b^	3 ± 2 ^d^
5	1032 ± 270 ^a b^	824 ± 166 ^a b^	130 ± 88 ^a b^	40 ± 20 ^a b^	1162 ± 337 ^a b^	877 ± 193 ^a b^	90 ± 5 ^a b^	96 ± 2 ^a b^	10 ± 5 ^a b^	4 ± 2 ^d^

^a^ Different from the other conditions (*p* < 0.05); ^b^ Different from all steps (*p* < 0.05); ^c^ Different from steps 3, 4, and 5 (*p* < 0.05); ^d^ Different from step 5 (*p* < 0.05).

**Table 3:** Loading values of the studied parameters in the first two principal components of the set of observations.

Parameter	Loading Values	
	Principal Component 1	Principal Component 2
Shoulder to hand distance	0.77	0.44
Stroke frequency	0.67	−0.25
Stroke length	−0.13	0.44
Active drag	0.84 *	0.45
Power to overcome drag	0.88 *	0.37
Speed-specific drag	0.41	0.80 **
Arm stroke efficiency	−0.87 **	−0.12
External mechanical power	0.92 **	0.33
Power wasted to the water	0.94 *	0.30
Aerobic metabolic power	0.94 *	0.07
Anaerobic metabolic power	0.77	−0.59
Total metabolic power	0.96 **	−0.09
Oxygen uptake	0.88 *	0.15
Blood lactate concentration	0.70	−0.64
Energy cost	0.93 **	−0.10
Aerobic contribution	−0.61	0.71
Anaerobic contribution	0.61	−0.71

* Redundant parameters with significant eigenvalues that were not included in the prediction model; ** Parameters selected for the prediction model.
